# Evolutionary history of Serpulaceae (Basidiomycota): molecular phylogeny, historical biogeography and evidence for a single transition of nutritional mode

**DOI:** 10.1186/1471-2148-11-230

**Published:** 2011-08-04

**Authors:** Inger Skrede, Ingeborg B Engh, Manfred Binder, Tor Carlsen, Håvard Kauserud, Mika Bendiksby

**Affiliations:** 1 Microbial Evolution Research Group (MERG), Department of Biology, University of Oslo, P.O. Box 1066 Blindern, N-0316 Oslo, Norway; 2 Department of Biology, Clark University, Worcester, Massachusetts 01610, USA; 3 National Centre for Biosystematics, Natural History Museum, University of Oslo, P.O. Box 1172 Blindern, N-0318 Oslo, Norway

## Abstract

**Background:**

The fungal genus *Serpula *(Serpulaceae, Boletales) comprises several saprotrophic (brown rot) taxa, including the aggressive house-infecting dry rot fungus *Serpula lacrymans*. Recent phylogenetic analyses have indicated that the ectomycorrhiza forming genera *Austropaxillus *and *Gymnopaxillus *cluster within *Serpula*. In this study we use DNA sequence data to investigate phylogenetic relationships, historical biogeography of, and nutritional mode transitions in Serpulaceae.

**Results:**

Our results corroborate that the two ectomycorrhiza-forming genera, *Austropaxillus *and *Gymnopaxillus*, form a monophyletic group nested within the saprotrophic genus *Serpula*, and that the *Serpula *species *S. lacrymans *and *S. himantioides *constitute the sister group to the *Austropaxillus*-*Gymnopaxillus *clade. We found that both vicariance (Beringian) and long distance dispersal events are needed to explain the phylogeny and current distributions of taxa within Serpulaceae. Our results also show that the transition from brown rot to mycorrhiza has happened only once in a monophyletic Serpulaceae, probably between 50 and 22 million years before present.

**Conclusions:**

This study supports the growing understanding that the same geographical barriers that limit plant- and animal dispersal also limit the spread of fungi, as a combination of vicariance and long distance dispersal events are needed to explain the present patterns of distribution in Serpulaceae. Our results verify the transition from brown rot to ECM within Serpulaceae between 50 and 22 MyBP.

## Background

Studying fungal distributions in an evolutionary context is relatively new, mainly due to the long-standing perception that fungi are more or less free from dispersal barriers, and that fungal distributions are primarily controlled by the distribution of hosts and substrata (see [1]). However, this notion of "cosmopolitan species" has recently been challenged by molecularly based studies [2-4]. Fungal distributional patterns can be complicated by the fact that cryptic species and polyphyly often are revealed in molecular based fungal studies. Thus, molecular phylogenetic approaches have proven important for the identification of unique lineages with restricted distributions [3]. Although it is obvious that hosts impose some restriction on the distribution of their parasite or symbiont (e.g. [5]), Taylor et al. [3] argue that very few (if any) organisms can be considered free of entanglement with other organisms. In fact, Parrent et al. [6] suggested that high levels of host specificity increase the effect of isolation-by-distance.

Vicariance versus long distance dispersal are two alternative explanations to widely disjunct distributions, and have been identified to operate in most groups of organisms [7,8]. It is becoming increasingly accepted that the same barriers to dispersal operate in fungi as in other groups of organisms [1,2]. Vicariance has given explanatory power for disjunct distributions in some extomycorrhizal fungi such as pacific boletes [9] and truffle-formin fungi in the Hysterangiales [10], while dispersal best explains the distribution of the wood-rot fungi *Ganoderma *[11].

Molecular dating makes it possible to relate organismal evolution to major ecological and geological events. The combination of fossils that correlate to divergence events in organismal phylogenies with increasingly realistic models of molecular evolution has increased the accuracy of molecular dating [12]. Reliable fossils are preferred for calibration of molecular clocks. However, dated geographic and ecological events as well as age estimates of symbiotic or parasitic partners have also been used [13-15], either in combination with fossil calibration or as the best available alternative when no reliably determined and correctly assigned fossils exist. Unfortunately, the fossil record of fungi is sparse [12], and several studies have attempted to date evolutionary splits of fungi using various calibration strategies (e.g. [15-20]). An additional complicating factor in molecular dating is between-lineage rate heterogeneity of molecular evolution, which has been extensively documented in fungi (see [12] and references therein). However, methods have been developed that account for rate heterogeneity by various sophisticated relaxed clock models (e.g. BEAST [21]).

In recent times, the fungal tree of life has been largely redrawn by molecular phylogenetics. This has in turn improved our understanding of how different nutritional modes have evolved [22-24]. The Agaricomycetes is a class in the fungal kingdom that contains most of the forest floor mushroom-forming fungi [25]. Currently saprotrophy (possibly white rot) is suggested to be the plesiomorphic state of the Agaricomycetes [26], while brown rot and ectomycorrhiza have evolved several times independently from the ancient white rot state. Ectomycorrhizal (ECM) fungi form mutualistic interactions between plant roots and fungal mycelia. The symbiosis provides the plant with important nutrients such as phosphorous and nitrogen, and the fungi receive photosynthetic products in return [27,28]. Mycorrhizal symbioses are ubiquitous in natural plant environments [29-31]. Saprotrophy by white rot decomposes lignocellulose and utilize all parts of the wood. Brown rot, on the other hand, only deforms the lignin to get access to the cellulose and hemicelluose, which are then efficiently depolymerized and absorbed by the fungus [32,33].

Recent work shows that ECM is a nutritional mode that has evolved several times independently in both plants and fungi (both Basidiomycota and Ascomycota) [28,34]. Hibbett and Matheny [26] concluded that there have been many independent origins of ECM associations of Agaricomycetes with both Angiosperms (at least 8) and Gymnosperms (at least 6-8). The monophyletic basidiomycete order Boletales comprises various nutritional modes, including ECM, saprotrophy and parasitism [35,36]. Brown rot has been suggested as the plesiomorphic state for the Boletales [37].

The genus *Serpula *(Pers.) Gray in the Boletales consists of saprotrophic taxa that mainly degrade conifer substrates [37]. *Serpula *species produce annual, brownish, and resupinate fruitbodies (flat structures adhered to a woody or soil substrate with a morphological cariable sexual spore-producing surface; in the case of *Serupla *this spore producing surface is wrinkled or merulioid) [38,39]. Many *Serpula *species appear frequently on structural timbers in buildings, including *S. himantioides *(Fr.) P. Karst., *S. incrassata *(Berk. & M.A. Curtis) Donk and *S. lacrymans *(Wulfen) J. Schröt [40]. *Serpula lacrymans *is very aggressive in buildings and produces what is commonly known as dry rot (ref). The taxonomic circumscriptions of *Serpula himantioides *and *S. lacrymans *have varied through time: they are lumped as one based on morphology [41] or recognized as two separate species based on mating experiments [42]. The morphotaxon *S. lacrymans *includes two varieties. One of them (var. *shastensis *Harmsen) seems to have a natural distribution in the Cascade Range in western North America, while the other (var. *lacrymans *[Wulfen] J. Schröt.) has a natural distribution in northeast Asia from where it has spread to most continents and colonised buildings (see [43] and references therein). The morphotaxon *S. himantioides *includes multiple (at least five) cryptic species [44,45], most of them with a primary affinity to southern South America and North America. *Serpula incrassata *seems to have a strictly North American distribution [41]. A less studied species, *Serpula puverulenta *(Fr.) Bondartsev, which also appears in houses, has at least a European and probably also a North American distribution [46,47]. *Serpula similis *(Berk. & Broome) Ginns is known from tropical regions in Africa and southeast Asia, where it is sampled on bamboo and hardwoods [48,49].

Molecular phylogenetic analyses have indicated that the ECM-forming genera *Austropaxillus *Bresinsky & Jarosch and *Gymnopaxillus *E. Horak are nested within *Serpula*, and the family Serpulaceae Jarosch & Bresinsky was described comprising these three genera [50]. *Austropaxillus *species have fruiting bodies with a stipe (stem) and pileus (cap), and a bear a gilled hymenophore (spore-producing surface). In contrast, *Gymnopaxillus *encompasses truffle-like hypogeous (under ground) fruit bodies [51]. Due to morphological and ecological similarities, these genera were earlier included in *Paxillus *Fr. [52]. *Austropaxillus *and *Gymnopaxillus *form ECM with roots of *Nothofagus *and *Eucalyptus *trees and are known only from the temperate Southern Hemisphere.

The aim of the present study was threefold. First, we wanted to further investigate phylogenetic relationships within Serpulaceae (previously described in [37,52]), in particular the relationship between the saprotrophic *Serpula *and the mycorrhiza-forming *Austropaxillus *and *Gymnopaxillus*. Secondly, we wanted to relate major Serpulaceae divergence events and ecological transitions to geologic and climatic conditions of the past. Finally, we wanted to reveal how many times the transition from brown rot to mycorrhizal symbiosis has occurred in Serpulaceae including reconstructing its ancestral biographical range within a historical geological context. We addressed these issues using a molecular phylogenetic approach, including Bayesian age estimation and various ancestral area analyses, on a multi-locus dataset.

## Methods

### Taxon sampling

We generated DNA sequence data from 30 accessions of five *Serpula *species and six accessions representing six species of *Austropaxillus *(Additional file 1: Specimens included in this study). In the phylogenetic analysis, we included an extensive set of outgroups from both the Boletales (41 accessions) and its sister group the Atheliales, the latter used for rooting purposes (Dataset1). For the purpose of dating, we reduced the boletalean outgroup to 24 accessions, expanded the athelialean outgroup to seven accessions, and included 1-24 accessions from each of nine additional fungal orders (Dataset2). Sequences not generated for the present study were taken from Binder et al. [53] and Jarosch and Bresinsky [50]. We used a total of 79 accessions for the phylogenetic analyses (Dataset1) and 109 accessions in the dating analysis (Dataset2). A separate phylogenetic analysis was performed on a reduced dataset of 39 accessions (Serpulaceae and a single outgroup taxon; Dataset3) to depict the phylogenetic position of *Gymnopaxillus*, for which only sequences of a single genetic region are available. All included accessions are listed in Additional file 1.

### DNA extraction, PCR amplification and DNA sequencing

A small amount of fungal tissue was homogenized on a Mixer Mill (MM301, Retsch GmbH & Co., Haan, Germany) before we extracted total DNA following the 2% CTAB miniprep method described by Murray and Thompson [54] with minor modifications from Gardes and Bruns [55]. We dissolved the dried DNA pellet in 100 μL milli-Q H_2_O, and used further dilutions for the molecular work. We have deposited the DNA aliquots in the fungal DNA collection at the Institute of Biology, UoO, Norway.

Five nuclear DNA regions were amplified: the nuclear ribosomal large subunit (LSU) using the primers LR0R/LR5 [56], the nuclear ribosomal small subunit (SSU) using the primers PNS1/NS41 [57], the nuclear ribosomal 5.8S using the primers ITS5 and ITS4 [58], a fragment of the gene that encodes the second largest subunit of the RNA polymerase II (*rpb*2) using the primers b3.1F and b6R2 [59], and the translation elongation factor 1α (*tef *1) using the primers EF595F and EF1160R [60].

We generated amplicons using the following PCR protocol: 4 min at 94°C; followed by 35 cycles of 25 s at 94°C, 30 s at 56°C (*rpb*2 53°C; ITS and *tef *154°C), and 60 s (30 s ITS and *tef *1) at 72°C; followed by a 10 min extension at 72°C and an indefinite hold at 4°C. For all PCR reactions we used the PuReTaq Ready-To-Go™ PCR Beads (GEhealthcare, Waukesha, WI) in a 25 μl reaction on an Eppendorf thermocycler (Mastercycler, Hamburg, Germany). We purified the PCR-products using 2 μl 10 times diluted ExoSAP-IT (GEhealthcare) to 8 μl PCR product, incubated at 37°C for 45 minutes followed by 15 minutes at 80°C. Cycle sequencing was performed by the CEES ABI-laboratory http://www.mn.uio.no/bio/english/research/about/infrastructure/abi-lab/index.html using the ABI BigDye Terminator sequencing buffer and v3.1 Cycle Sequencing kit (Life Technologies, Carlsbad, CA). Sequences were processed on an ABI 3730 DNA analyser (Life Technologies). Sequences were assembled and edited using BioEdit 7 [61]. All sequences have been deposited in GenBank, and accession numbers are given in Additional file 1.

### Datasets

We aligned the sequences manually using BioEdit 7. We assembled three datasets: Dataset1, consisting of a 2856 basepair (bp) long alignment that includes five genetic regions (nuclear ribosomal LSU, SSU and 5.8S, *rpb*2 and *tef *1), available as Additional file 2; Dataset2, consisting of a 6172 bp long alignment including longer stretches of the above-mentioned markers, available as Additional file 3; Dataset3, consisting of a 918 bp long alignment including LSU only and a total of 38 Serpulaceae accessions (incl. *Gymnopaxillus*) and *Bondarcevomyces *for rooting, available as Additional file 4. Due to restricted sequence data, we did not include *Gymnopaxillus *in the final analyses, but Dataset3 was analyzed to confirm the phylogenetic position of *Gymnopaxillus *within Serpulaceae and together with *Austropaxillus*.

### Phylogenetic analyses

Dataset1 and 2 were analyzed using both Maximum Parsimony (MP) and Bayesian Inference (BI) phylogentic methods. Dataset3 was analyzed using MP only. We performed preliminary MP phylogenetic analyses on each DNA region separately to control visually for between-region incongruence prior to concatenation and final phylogenetic analyses. For the MP analyses of the concatenated datasets and Dataset3 we used TNT [62]. We performed heuristic searches with 2000 random addition sequences and TBR branch swapping, saving ten trees per replication. The resulting trees were swapped with TBR saving up to 10 000 trees. We set the collapsing rule to minimum length = 0 and random seed was set to "time". Jackknife [63] resampling was performed with 2000 replicates (10 random entry orders and 10 trees saved each repetition), 36% deletion, absolute frequencies as output, and a cut-off value of 50%.

The best-fit evolutionary models were estimated using the Akaike information criterion (AIC) and the software MrModeltest 2.3 [64] at the Bioportal http://www.bioportal.uio.no. We divided the datasets (Dataset1 and Dataset2) into three partitions: (1) the ribosomal regions (SSU, LSU and 5.8S; collectively nrDNA), (2) *rpb*2, and (3) *tef *1. The best-fit models for these three parts were: GTR+I+G for the ribosomal regions, HKY+I+G for *rpb*2, and SYM+I+G for *tef *1.

For the BI analyses, we analyzed a partitioned (according to the above established parts) concatenated alignment of all five nuclear regions using MrBayes 3.1.2 [65,66] at the Bioportal. Bayesian posterior probabilities were determined twice by running one cold and three heated chains for six million generations, saving trees every 1000th generation. To test whether the Markov Chain converged, we monitored the standard deviation of split frequencies (SDSF), and discarded as burn-in the generations prior to the point when the SDSF fell below 0.01 when comparing two independent runs. A 50% majority rule consensus tree was used to calculate posterior probabilities.

### Divergence time estimation

We used Dataset2 (five DNA regions and 109 accessions) and the computer program Bayesian Evolutionary Analysis Sampling Trees (BEAST) 1.6.1 [21] for estimating the posterior probability distribution of divergence times in Serpulaceae. BEAST co-estimates phylogeny and divergence times [67]. Details about the Bayesian approach used in BEAST have been described by others [12].

**Figure 1:**
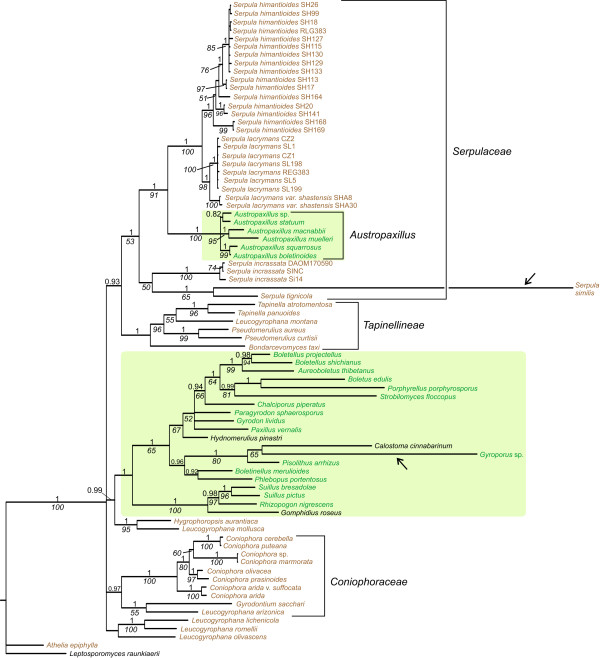
**Serpulaceae phylogeny**. The 50% majority rule consensus tree from a Bayesian analysis of a concatenated data set consisting of parts of nuclear ribosomal SSU, LSU, and 5.8S, the second largest subunit of the RNA polymerase II (*rpb*2), and the translation elongation factor 1α (*tef *1). Bayesian posterior probabilities (above branches) of more than 0.9 and parsimony Jackknife values (below branches) above 50% are superimposed on the phylogenetic tree. Saprotrophic taxa are in brown whereas ECM taxa are in green with a shaded green box.

Xml-files for the BEAST analyses were constructed using BEAUti 1.6.1 (BEAST package). We analyzed the data under four different treatments; different clock models (strict versus uncorrelated lognormal relaxed [UCLD]), and different prior distributions for the calibrations (exponential versus lognormal). The xml-files of uncorrelated lognormal relaxed clock with exponential and lognormal prior distributions are available as Additional file 5 and Additional file 6, respectively. Because among branch rate heterogeneity within the data was high, we also performed estimations without long-branch taxa (indicated by arrows in Figure 1). However, as divergence time estimates from the analyses with and without long-branch taxa were highly similar, only results from the analysis including the long-branch taxa is presented (see Results). We partitioned the data according to marker and codon position (only the two coding genes; [1+2],3). Partitioning of data into markers and appropriate codon usage has been shown to produce shorter node heights than unpartitioned analyses [68]. A Yule tree prior was employed in all runs, which assumes a constant speciation rate for each branch in the tree. A lognormal distribution was used for the priors for all calibrated nodes because it fixes the minimum age and allows the maximum age to be sampled following a lognormal distribution with no hard upper limit.

**Figure 2:**
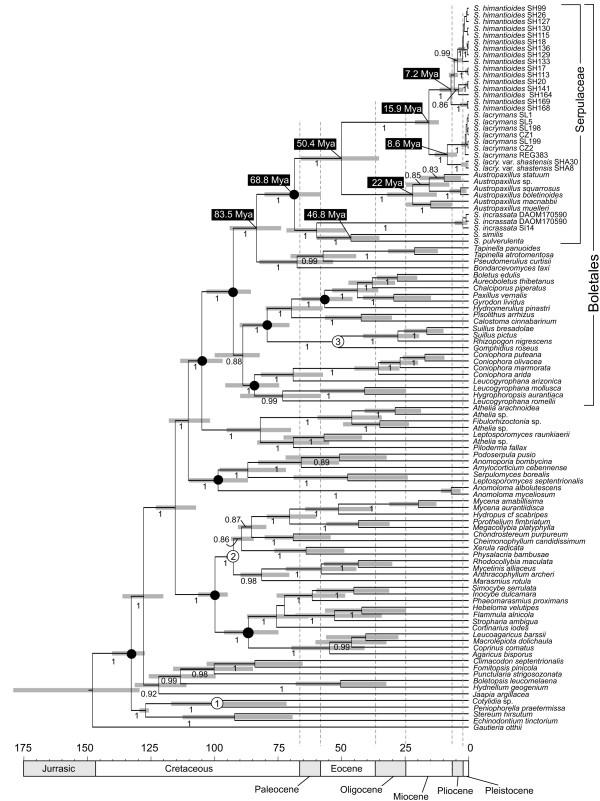
**Molecular dating analysis**. The MCC chronogram of an Agaricomycetidae subset, with main focus on Boletales taxa, obtained from divergence time estimation using BEAST. Grey bars denote the 95% highest posterior density (HPD) intervals of the posterior probability distribution of node ages. Calibrated nodes are numbered (see text for details). Bayesian posterior probabilities of more than 0.8 are indicated on branches. Numbers on the scale axis are in millions of years before present (MyBP). Black dots at nodes indicate monophyly-contraints used in the BEAST analysis.

Since there are no known fossils of Serpulaceae we used an extensive outgroup for which fossils are available. We included three primary calibration points (corresponding to the numbered nodes in Figure 2): (1) *Quatsinoporites cranhamii *Smith, Currah and Stockey from the Early Creatceous (Barremian) [69]; (2) *Archeomarasmius leggetti *Hibbett, Grimaldi and Donoghue from the early Late Cretaceous [70,71]; and, (3) a permineralized "sulloid" ECM associated with *Pinus *roots of at least 50 million years [72]. We used the following lognormal settings (Mean in Real Space): (1) logmean = 2.0, logstdev = 0.5, and offset = 125.0; (2) logmean = 2.5, logstdev = 0.5, and offset = 90.0; and, (3) logmean = 1.5, logstdev = 0.5, and offset = 50.0. The exponential settings were: (1) mean = 3.0 and offset = 130.0; (2) mean = 3.2 and offset = 90.0; and, (3) mean = 2.0 and offset = 50.0. These lognormal/exponential settings correspond approximately to the following median and 95% HPD (square-bracketed; numbers rounded off to nearest million): (1) 127 [126-129]/132 [130-139]; (2) 92 (2) 92 [91-95]/92 [90-99]; and (3) 51 [50-53]/51 [50-56]. BEAST may get stuck on local optima because it does not employ a coupled MCMC. Thus, ten additional monophyletic sets of taxa (indicated with black dots on Figure 2) were predefined in order to obtain a topology that corresponds to the phylogeny obtained from a previous study [53] that used a more robust phylogenetic method (e.g. MrBayes) and more taxa. The Markov chains were run for ten million generations, sampling and saving every 1000th tree. Convergence of the chain to stationary distributions was controlled by inspection of the MCMC samples (Trace) in each analysis using the program Tracer 1.5 (BEAST package), and 1000 trees (i.e. 10%) were discarded as burn-in prior to summarizing the posterior distribution of trees and indentifying the maximum clade credibility (MCC) tree using TreeAnnotator 1.6.1 (BEAST package). Each treatment was analyzed five times to determine if the independent runs converged on the same posterior distribution. In order to obtain effective sample sizes (ESS) of more than 100 for all parameters, we combined the log output files from separate runs with the same treatment using LogCombiner 1.6.1 (BEAST package). To compare the different clock treatments, we monitored the "CoefficientOfVariation" for each partition in the relaxed clock analyses, and discarded a strict clock evolution if the distribution did not bump up against zero.

### Ancestral area analyses

We predefined unit areas based on present-day distributions of Serpulaceae taxa. The following seven unit areas were used: (A) western North America; (B) eastern North America; (C) western Eurasia; (D) eastern Eurasia; (E) southern South America; (F) Australia + New Zealand; and, (G) Old World Tropical. For each terminal taxon we coded the unit area(s) in which it presently occurs. The variety *Serpula lacrymans *var. *shastensis *has been recorded once in the Caucasus [43], outside its range as defined in this study. However, this observation has not been confirmed by other studies.

We used two alternative event-based ancestral area methods: (1) a Bayesian approach to dispersal-vicariance analysis (DIVA [73]) as implemented in the computer software Reconstruct Ancestral States in Phylogenies (RASP [74]) and, (2) the dispersal-extinction-cladogenesis analysis (DEC [75]) implemented in the computer program LAGRANGE. The Bayesian approach in RASP is built on the source code of MrBayes 3.1.2, and is a generalization of the methods used in Olsson et al. [76] and Sanmartin et al. [77]. We chose the F81 model for the Bayesian MCMC analyses, allowing for different rates of change among ancestral areas. The Bayesian posterior probabilities were determined twice by running 10 chains fifty thousand generations, saving reconstructions every 100th generations.

The ancestral area analyses were conducted on the posterior distribution of dated Serpulaceae trees estimated from BEAST. RASP analyses were carried out using 10^3 ^randomly selected trees from BEAST.

The DEC analyses in LAGRANGE enables maximum likelihood estimation of range inheritance scenarios at cladogenesis events by modelling transitions between discrete states (ranges) along phylogenetic branches as a function of time. The DEC script was produced using a web-based analysis configuration tool available from http://www.reelab.net/lagrange/configurator/index.

## Results

### Phylogeny

In the MP analysis of Dataset1, we obtained 469 most parsimonious trees (MPTs) of 4485 steps and with a reconciled consistency index (RC) of 0.2 and a homoplasy index (HI) of 0.69. Our results from the BI and MP analyses were largely congruent, but variously resolved. The Bayesian 50% majority rule consensus tree is presented with branch support (posterior probabilities [PP] and parsimony jackknifing [JK]) superimposed in Figure 1. Results render Serpulaceae monophyletic with Tapinellineae as phylogenetic sister (Figure 1). The Serpulaceae/Tapinellineae group consists of brown rot species except for the *Austropaxillus *clade. The brown rot species belonging to *Coniophora *and Coniophoraceae appear as a monophyletic group separated from the Serpulaceae/Tapinellineae clade (Figure 1).

The saprotrophic *Serpula himantioides *and *S. lacrymans *appear as sister taxa in the phylogeny and together form a monophyletic group (Figure 1). *Serpula lacrymans *is further subdivided in two lineages representing the two described varieties, var. *lacrymans *and var. *shastensis *(see [43]). *Austropaxillus *forms a monophyletic sister to the *S. lacrymans/S. himantioides *group that makes *Serpula *paraphyletic as currently circumscribed (Figure 1). A clade comprising the saprotrophic species *Serpula incrassata*, *S. pulverulenta *and *S. similis *is recovered as sister to all other Serpulaceae (Figure 1). The branch leading to *S. similis *is very long, indicating an increased rate of molecular evolution in this lineage.

The MP analysis of Dataset3 produced 16 MTPs of 311 steps, an RC of 0.90, and an HI of 0.06. The strict consensus tree with JK values superimposed is presented in the Additional files. A monophyletic *Gymnopaxillus *group renders *Austropaxillus *paraphyletic (Additional file 7, Figure. S1).

### Divergence time estimation

**Figure 3:**
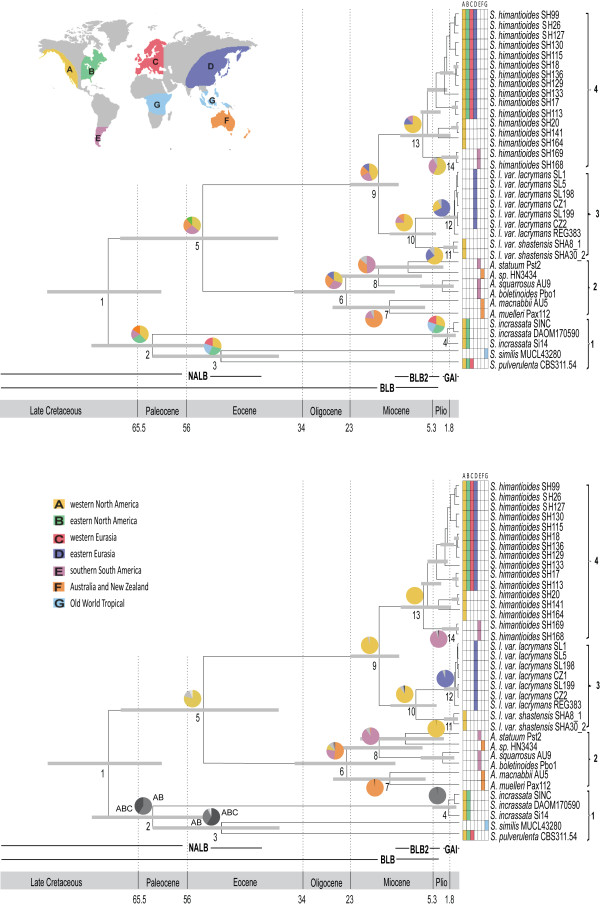
**Serpulaceae chronogram**. Out-cut of Serpulaceae from the chronogram presented in Figure. 2 (the maximum clade credibility tree obtained from divergence time estimation using BEAST). Grey bars denote the 95% highest posterior density (HPD) intervals of the posterior probability distribution of node ages. Nodes that are referred to in the text and in Table 1 are numbered and clades that are referred to in the text are numbered 1-4 to the right. Numbers on the scale axis are in millions of years before present (MyBP). Pie diagrams show the ancestral distributions estimated for internal nodes using a) DEC (LAGRANGE) and b) RASP. Only ancestral areas with probability > 0.01 are shown on the figure. Grey colour on pie diagrams indicate combined ancestral areas, and for nodes where a combined ancestral area is estimated as highly probable the combination is written next to the pie diagram. Abbreviations: Pli = Pliocene, NALB = North American landbridge, BLB = Bering landbridge, BLB2 = Bering landbridge 2, and GAI = Great American interchange.

The posterior distribution of the "CoefficientOfVariation" suggests rejection of a strict clock for all gene partitions. Relaxed (UCLD) clock analyses with lognormal versus exponential priors for the calibrations estimated rather similar age estimates of Serpulaceae divergences (Table 1). The maximum clade credibility (MCC) tree obtained from the relaxed clock/lognormal treatment is presented in Figure 2. A subsection of this chronogram, including Serpulaceae only, is presented in Figure 3. Mean nodal age estimates for numbered nodes in Figure 3 and their respective 95% highest posterior density (HPD) intervals are reported in Table 1.

**Table 1:** Results from phylogenetic, dating and ancestral area analyses

Node	Branch support ^1^	Node age lognormal ^2^	95% HPD lognormal	Node age exponential^2^	95% HPD exponential	RASP	DEC (LAGRANGE)
1	1/51	68.75	57.93-80.08	69.46	58.36-81.25	--	--
2	0.66/--	60.07	48.23-71.46	60.27	48.65-72.11	AB (0.576), ABC (0.400)	A (0.029), B (0.022), E (0.014), F (0.012)...
3	1/61	56.80	34.97-59.63	46.64	33.5-59.03	ABC (0.551), AB (0.316), BC (0.032), AC (0.031), B (0.018), A (0.018), ABCG (0.016)	A (0.035), B (0.033), G (0.027), C (0.023)...
4	1/100	2.61	0.45-5.41	2.67	0.62-6.24	AB (0.982), ABC (0.0163)	A (0.035), B (0.033), G (0.027), C (0.023)...
5	1/90	50.44	35.15-65.82	50.72	33.61-67.35	A (0.706), AB (0.125), ACB (0.038), C (0.018), AE (0.014), ABC (0.010)	A (0.031), E (0.024), F (0.021), B (0.010)
6	1/100	22.03	12.40-31.92	22.33	13.09-31.68	F (0.494), E (0.241), A (0.092), EF (0.079), AF (0.030), AE (0.015)	A (0.029), E (0.028), F (0.025), D (0.013)...
7	1/95	15.22	6.63-24.43	15.40	7.48-24.56	F (0.987)	F (0.198), E (0.044), EF (0.017)
8	--/100	15.71	7.46-24.47	15.87	8.23-25.38	E (0.994), EF (0.078), F (0.017)	E (0.130), F (0.096), EF (0.037)
9	1/100	15.88	11.63-21.00	16.25	11.53-21.06	A (0.941), AE (0.025), AD (0.011)	A (0.044), E (0.024), F (0.019), D (0.013)...
10	1/98	8.60	4.45-13.23	8.81	4.63-13.65	A (0.905), AD (0.071), D (0.015)	A (0.140), D (0.026), E (0.022)
11	1/100	1.19	0.05-2.91	1.32	0.11-3.12	A (0.997)	A (0.251), D (0.079), AD (0.035)
12	1/100	1.44	0.45-2.88	1.49	0.22-2.94	D (0.941), AD (0.055)	D (0.260), A (0.094), AD (0.031)
13	1/92	7.29	4.29-11.31	7.70	4.31-11.67	A (0.883), AE (0.090), E (0.019)	A (0.140), D (0.026), E (0.022)

Results from various analyses of Dataset2 (node numbers refer to Figure. 3): branch support, mean node ages, the 95% highest posterior density values (HPD; depicted as grey bars in Figure. 3) from analyses using a relaxed clock with lognormal and exponential settings, and inferred ancestral areas at internal nodes (RASP) and branches (DEC; relative probability of an area reported for branch leading to numbered node). Areas with a relative probability of > 0.01 in the DEC and RASP analyses are reported. Unit areas: A = western North America, B = eastern North America, C = western Eurasia, D = eastern Eurasia, E = southern South America, F = Australia + New Zealand, G = Old World Tropical.

^1^Bayesian posterior probability to the left and parsimony jackknife support to the right hand side of slash.

^2^Estimated age of divergence events in millions of years before present (depicted as a chronogram in Figure. 2).

*Not present for maxareas = 2

According to the estimated divergence times, Serpulaceae diverged from the Tapinellineae during the Late Cretaceous, and the mean crown-group age of Serpulaceae was estimated to about 64 (80-58) MyBP (Figure 2; Table 1; Figure 3, clade 1). The three *Serpula *species, *S. incrassata*, *S. similis *and *S. pulverulenta *(Figure 3, clade 2) appear to be the oldest species within Serpulaceae, with divergences among sampled species evident from the Late Cretaceous to Early Eocene (71-48 MyBP).

The divergence between the ECM group and the brown rot species (*Austropaxillus *vs. *S. lacrymans *and *S. himatioides*) is estimated to have happened between the Late Cretaceous to Late Eocene (66-35 MyBP; Figure 3, node 5). The posterior mean crown-group age of *Austropaxillus *is approximately 22 (32-12) My (Figure 3, node 6). Subsequent speciation events of sampled *Austropaxillus *species seem to suggest a fairly constant rate of speciation. The divergence of the two widely distributed *Serpula lacrymans *and *S. himantioides *is estimated to have occurred in Miocene; the mean age estimate being 16 MyBP (Figure 3, node 9). Intraspecific diversification leading to varieties and cryptic species within *S. lacrymans *and *S. himantioides *is estimated to have occurred during the Miocene and Pliocene (Figure 3).

### Ancestral area analyses

The inferred historical biogeographic scenarios from analyses using DEC (from LAGRANGE) and RASP are summarized in Table 1 and Figure 3. The inferred ancestral areas at internal nodes estimated using the Bayesian RASP correspond largely to the results obtained from the maximum likelihood estimates in DEC. RASP estimated more combined ancestral areas than DEC, and the RASP estimates have generally a higher relative probability for each ancestral area (Table 1).

The maximum likelihood reconstruction of ancestral areas for the internal nodes of Serpulaceae show that the most recent common ancestor (tmrca) of clades 2-4 (Figure 3, node 5) most likely appeared in western North America during the Paleocene-Eocene boundary. Range expansion of one daughter lineage, *Austropaxillus *(Figure 3, clade 2), into South America, Australia and/or New Zealand some time during the Eocene to Mid Miocene followed this. The divergence into one mainly southern South American clade (node 8) and one Australian/New Zealand clade (node 7) is estimated to have happened about 15 (24-7) MyBP. The second daughter lineage, tmrca of *S. himantioides *and *S. lacrymans *(Figure 3, clades 3-4, node 9), most likely occurred in western North America during the Early Eocene to Mid Miocene.

## Discussion

### Molecular phylogenetics of Serpulaceae

Our results based on five nuclear DNA regions corroborate the monophyly of Serpulaceae sensu Jarosch [50]. Moreover, a close relationship between the saprotrophic *Serpula *and the ectomycorrhizal *Austropaxillus *is supported, as already indicated in previous studies based on more restricted taxon samplings and fewer genetic markers [37,50]. *Gymnopaxillus *nested within the Australian *Austropaxillus *clade (Additional file 7, Figure. S1) in the nrLSU analysis. Traditionally, *Serpula *has been recognised as a member of Coniophoraceae [39,78]. Our molecular results clearly show that *Serpula *is only distantly related to *Coniophora*, supporting the treatment of Serpulaceae [50] as a separate family that comprises the three genera *Austropaxillus*, *Gymnopaxillus *and *Serpula*. That *Austropaxillus *and *Gymnopaxillus *are nested within *Serpula *suggests taxonomic changes are needed to accommodate this monophyly. Unfortunately we were unable to include samples from South American *Gymnopaxillus *species, so our results call for further analyses including more markers and samples from this group.

### Historical biogeography of Serpulaceae

Our results indicate a Late Cretaceous origin of extant Serpulaceae, between 94 and 74 MyBP (Figure 2) in temperate North America (Figure 3). The Gymnosperm genus *Pinus *is the main substrate of Serpulaceae in the Northern Hemisphere. *Pinus*, is estimated to have evolved during the Cretaceous, between 155 and 87 MyBP [79]. Thus the age of *Pinus *corresponds well with the timing of the evolution of Serpulaceae on *Pinus *wood. Moreover, the fossil record supports the presence of Pinaceae members in the high-latitude and high-altitude regions of North America during the early Cenozoic [72]. This supports our result, as the main host of Serpulaceae was available at the time of origin.

The initial divergence of extant *Austropaxillus *taxa into one mainly southern South American clade and one Australian/New Zealand clade about 22 (32-12) MyBP (Figure 3, node 6) must represent a long distance dispersal event, and is consistent with a biogeographical tract referred to by Crisp et al as a pan-temperate tract [80]. Nor does the relatively recent split between northern and southern hemisphere taxa in Serpulaceae support an ancient Gondwanan distribution of *Austropaxillus*. However, there have been fierce discussions of the time and climatic conditions of the Gondwanan break-up [81]. Land bridges have been suggested between Antarctica and Australia up until 28 MyBP [82,83]. During this period Antarctica was probably glaciated [81] and may have been too hostile for temperate fungi. Thus, even if we cannot rule out vicariance, we consider long distance dispersal a more plausible explanation. Moreover, the divergence times estimated within Serpulaceae can be expected to be somewhat younger due to the node density effect [84], supporting a long-distance dispersal event.

The divergence between *Austropaxillus macnabbi *from New Zealand and *A. muelleri *from Tasmania (Figure 3, node 7) must have resulted from a more recent dispersal event. New Zealand was separated from South America and Australia about 80 MyBP [82], i.e. about 60 My years before the split at node 7 (24-7 MyBP). Interestingly, the same recent divergence between species from New Zealand and Australia have also been found for *Nothofagus *[85], the host of *Austropaxillus*, and its fungal parasite *Cyttaria *[86]. Long distance dispersal is also suggested in order to explain the divergence between *Austropaxillus *sp. from Tasmania and *A. statuum *from southern South America (Figure 3). However, as the sister-relationship between the two species is weakly supported, more data is needed to confirm or reject this long distance dispersal hypothesis. Additional fungal disjunctions that are too young to be caused by Gondwanan breakup, and thus are explained by long-distance dispersal have been reported elsewhere (e.g. [10,11,18]).

Assuming western North America as the true ancestral area at node 5 (Figure 3), the presence of southern South American species in both clade 2 (*Austropaxillus*) and clade 4 (*S. himantioides*) must be explained by independent long-distance dispersal events. This can be concluded, because Laurasia (incl. North America) and Gondwana (incl. southern South America) were separated by the east-west Tethyan seaway from the latest Jurassic (c 150 MyBP) to the end of Pliocene (c 3 MyBP; [87]). This seaway represented a major dispersal barrier to most terrestrial organisms. Not until the establishment of the Great American Interchange (GAI; ca. 3 MyBP) was there land connection between North and South America (Figure 3). Although the two dispersals happened at different times, none correspond to time periods of land-connection between North and South America. Moreover, the large distributional gap between North American and southern South American sister-taxa of *Serpula *(see inset map in Figure 3) favours long distance dispersals to historically continuous distributions with subsequent vicariance. Still, the distinctness of the disjunct lineages indicates that such inter-area dispersals are not frequent. Nevertheless, historical long distance dispersal has been reported in several mycogeographic studies (e.g. [11,18,88]), and dispersal between northern and southern temperate regions of the world seems to have occurred in other fungal groups also (e.g. [10,20]) and plants [80].

All taxa represented in clade 3 (Figure 3) belong to the same morphospecies, *Serpula lacrymans*. The chronogram shows that the divergence of the two varieties occurred about 9 (13-4) MyBP (Figure 3, node 10). The presence of western North American and eastern Eurasian phylogenetic sister-taxa are indicative of a trans-Beringian distribution of their most recent common ancestor with subsequent vicariance (Figure 3). About 14-3.5 MyBP, there was continuous boreal forests across Beringia (Figure 3, BLB2; [89]). It seems likely therefore, that *S. lacrymans *had a continuous distribution from northern North America and into Eurasia and that var. *lacrymans *became differentiated from var. *shastensis *due to Beringan vicariance (Figure 3). Comparison of ancient permafrost-preserved (plant-parasitic) fungal DNA from north-eastern Siberia to modern tundra macrofungi indicate a shift in fungal diversity following the last ice-age [90], a scenario probably reiterated many times during the Pleistocene producing allopatric populations. Migration across a North Atlantic landbridge (NALB) has sometimes been used as an alternative explanation to Nearctic-Palearctic disjunctions, but this bridge disappeared by Mid Eocene (Figure 3; [89]) -- i.e. about 30 My before the estimated divergence of the two varieties. Historical Beringian distributions with subsequent vicariance and range expansion southwards have been shown also in *Amanita *[91] and *Tricholoma *[92], and we suspect that additional Beringian disjunctions would have been identified if the Holarctic was not treated as a single area in mycogeography (see e.g. [11,20]).

### Evolution of nutritional modes in Serpulaceae

According to our results, the transition to ECM has happened only once in Serpulaceae, in the lineage leading to *Austropaxillus *(Figure 1). Transition from a saprotrophic to ectomycorrhizal nutritional mode is a common ecological transition [28]. The common ancestor of *Austropaxillus *and *Gymnopaxillus *diverged from the saprotrophic clade about 50 MyBP. The temperature decline and a drier climate in the end of the Eocene and the onset of Oligocene may have promoted this transition from saprotrophy to mycorrhiza. When growing in nature, *S. lacrymans *mainly occurs on substrates in close contact with the ground (H. Kauserud, personal observations), and it seems to be a geophilous fungus that may transport water from the ground and into the substrate. Whether such an adaptation could be a first step towards mycorrhizal symbiosis is a subject for future investigation.

Our results indicate that the mycorrhizal *Austropaxillus/Gymnopaxillus *lineage diverged from the saprotrophic *S. lacrymans/S. himantioides *group about 50 (66-35) MyBP and that the radiation of extant *Austropaxillus/Gymnopaxillus *taxa commenced about 22 (32-12) MyBP (Figure 3). A more recent split between *Austropaxillus *and *S. lacrymans *(53.1-15 MyBp) was suggested in a recent paper analyzing a smaller dataset of genome-sequenced fungi [93]. The differences between these studies can probably largely be explained by taxon sampling. Because of the focus of this study, we have included more samples from the Serpulaceae compared to other fungal families; this could give a somewhat older age according to the node density effect [84] as argued above. Fossilized ECM demonstrates that ECM associations had evolved at least 50 MyBP, probably with a *Pinus*-host [94]. It has been suggested that the radiation of ECM fungi happened as the obligate ECM hosts (Pinaceae and Fagales) became dominant in temperate forests as a consequence of drying and cooling from the Late Eocene [16,20].

## Conclusions

Our analyses support the monophyly of Serpulaceae sensu Jarosch and a Late Cretaceous origin of the family. A single transition from brown rot to ECM occurred within the Serpulaceae. The dating analysis indicates that this transition happened between 50 and 22 MyBP. Most Serpulaceae divergences seem to have happened within continents (Australia, South American, and North America). A combination of long distance dispersals for the southern hemisphere taxa and vicariance for the northern hemisphere taxa is used to explain the distribution of extant Serpulaceae taxa. The dating analysis indicates that the divergence of the two varieties of *Serpula lacrymans *occurred between 13 and 4 MyBP due to Beringian vicariance.

## Authors' contributions

IS and MiB participated in analyzing the data and wrote most of the manuscript. IBE generated data, established alignments, participated in analyzing the data, and wrote parts of the manuscript. MaB generated data, established alignments and commented upon the manuscript. TC participated in analyzing the data and commented upon the manuscript. HK conceived of the study, and participated in its coordination and helped to draft the manuscript. All authors read and approved the final manuscript.

## Supplementary Material

Additional file 1 **Specimens included in this study**. A table including origin, Isolate ID and GenBank accession numbers for each specimen in the study. Click here for file

Additional file 2 **Allignment of Dataset1**. A 2856 bp allignment of the five nuclear regions 5.8S, nrLSU, nrSSU, *rpb2*, *tef1 *for 79 accessions of Serpulaceae taxa including an extensive boletalean outgroup (41 accessions) and two athelialean accessions for rooting the tree. Click here for file

Additional file 3 **Allignment of Dataset2**. A 6172 bp allignment of the five nuclear regions 5.8S, nrLSU, nrSSU, *rpb2*, *tef1 *for 109 accessions of Boletales and nine additional fungal orders. Click here for file

Additional file 4 **Allignment of Dataset3**. Allignment of the nuclear ribosomal LSU region for 39 accessions of the Serpulaceae and one outgroup. Click here for file

Additional file 5 **Xml file using an exponential prior distribution for Dataset2**. An .xml file created by BEAUti 1.6.1 for analyses of the data using an uncorrelated exponential relaxed molecular clock in BEAST. Click here for file

Additional file 6 **Xml file using a lognormal prior distribution for Dataset2**. An .xml file created by BEAUti 1.6.1 for analyses of the data using an uncorrelated lognormal relaxed molecular clock in BEAST. Click here for file

Additional file 7 **Serpulaceae nrLSU phylogeny including *Gymnopaxillus***. Strict consensus tree from a maximum parsimony analysis of the nuclear ribosomal LSU region of 38 Serpulaceae taxa. *Bondarcevomyces taxi *was used as outgroup. The 16 most parsimonious trees (MPTs) were 311 steps long and had a rescaled consistency index of 0.90 and homoplasy index of 0.06. Jackknife support values (2000 replicates) are superimposed on the branches, showing that the included *Gymnopaxillus *spp. (grey shading) are nested within *Austropaxillus *with high support. Click here for file
