# Supplementary material for: Evolutionary history of Serpulaceae (Basidiomycota): molecular phylogeny, historical biogeography and evidence for a single transition of nutritional mode
Source: BMC Evol Biol. 2011 Aug 4;11:230. doi: 10.1186/1471-2148-11-230 (PMC3199774; doi:10.1186/1471-2148-11-230)
Supplement: Additional file 1 — Specimens included in this study. A table including origin, Isolate ID and GenBank accession numbers for each specimen in the study. [file 1471-2148-11-230-S1.DOC]

**Additional files**

**Additional file 1 - Specimens included in this study.**

Origin, Isolate ID, and GenBank accession numbers are given when available.

| **Species** | **Isolate ID** | **Origin** | **5.8S** | **nuc-LSU** | **nuc-SSU** | ***rpb2*** | ***tef1*** |
| --- | --- | --- | --- | --- | --- | --- | --- |
| *Agaricus bisporus* | AFTOL-448 | USA, Pennsylvania | DQ404388 | AY635775 | AY787216 | AF107785 | GU187673 |
| *Amylocorticium cebennense* | HHB-2808 | USA, North Carolina, Skitty Creek Trail, Nantahala National Forest | GU187505 | GU187561 | GU187612 | GU187770 | GU187675 |
| *Anomoloma albolutescens* | L-6088 | USA, Colorado, Pyramid White River National Forest | GU187507 | GU187563 | GU187618 | GU187768 | GU187671 |
| *Anomoloma myceliosum* | MJL-4413 | Canada, British Columbia, Olive Lake, K. N. P. | GU187500 | GU187559 | GU187614 | GU187766 | GU187677 |
| *Anomoporia bombycina* | L-6240 | USA, Colorado, Arapaho National Forest | GU187508 | GU187564 | GU187611 | GU187765 | GU187674 |
| *Anthracophyllum archeri* | AFTOL-973 | Australia, Karri Valley, southwest Western Australia | DQ404387 | AY745709 | DQ092915 | DQ385877 | DQ028586 |
| *Athelia arachnoidea* | CBS418.72 | The Netherlands | GU187504 | GU187557 | GU187616 | GU187769 | GU187672 |
| *Athelia epiphylla* | FP-100564 | USA, Maryland, Patuxent Wildlife Research Refuge | GU187501 | GU187558 | GU187613 | GU187771 | GU187676 |
| *Athelia* sp. | FP-133442 | USA, Oregon, Mary's Peak, Siuslaw National Forest | GU187503 | GU187560 | GU187615 | GU187772 | GU187679 |
| *Athelia* sp. | HHB-15599 | USA, Alaska, Serpentine Hot Springs, Bering Land Bridge Nat'l Preserve | GU187502 | GU187565 | GU187619 | GU187767 | GU187678 |
| *Athelia* sp. | L-10567 | USA, Oregon, Gold Beach | GU187537 | GU187592 | GU187645 | GU187802 | GU187739 |
| *Aureoboletus thibetanus* | AFTOL-450 | China, Yunnan, Kunming | DQ200917 | AY700189 | AY654882 | DQ366279 | DQ029199 |
| *Austropaxillus* sp. | HN3434 | Australia: Tasmania, Waldheim forest walk, Cradle Mt. National Park | DQ534572 | DQ534670 | DQ534673 | — | — |
| *Austropaxillus macnabbii* | AU5 | New Zealand, Springs Junction, Lake Daniells Track | HM135656 | HM135706 | HM135787 | — | HM135557 |
| *Austropaxillus squarrosus* | AU9 | Argentina, Rio Negro, Depto. Bariloche, Río Manso Inferior | HM135657 | HM135707 | HM135788 | — | HM135558 |
| *Austropaxillus muelleri* | Pax112 | Australia, Tasmania | HM135658 | HM135708 | — | — | HM135559 |
| *Austropaxillus statuum* | Pst2 | Chile, Valdivian forest | HM135659 | HM135709 | HM135789 | HM135764 | HM135560 |
| *Austropaxillus boletinoides* | Pbo1 | Chile, Region Monumento National Parque Contulmo | HM135660 | HM135710 | — | HM135765 | HM135561 |
| *Boletellus projectellus* | AFTOL-713 | USA, Massachusetts, Cape Cod, Wellfleet, Marconi Station Rd near Atlantic White Cedar Swamp | AY789082 | AY684158 | AY662660 | AY787218 | AY879116 |
| *Boletellus shichianus* | AFTOL-532 | China, Yunnan, Kunming | DQ200921 | AY647211 | AY657011 | DQ366280 | DQ408145 |
| *Boletinellus merulioides* | AFTOL-575 | USA, Massachusetts, West Brookfield, Rock House | DQ200922 | AY684153 | AY662668 | DQ366281 | DQ056287 |
| *Boletus edulis* | Be3 | Germany, Bavaria, Regensburg | AY680988 | AF456816 | DQ534675 | GU187774 | GU187682 |
| *Boletopsis leucomelaena* | AFTOL-1527 | USA, Massachusetts, deCordova Museum, Flint's Pond, Lincoln | DQ484064 | DQ154112 | DQ435797 | GU187820 | GU187763 |
| *Bondarcevomyces taxi* | Dai2524 | China, Jilin Province, Antu County, Changbai Shan Nat. Res. | DQ534575 | DQ534672 | DQ534677 | — | GU187683 |
| *Calostoma cinnabarinum* | AFTOL-439 | USA, Massachusetts, Blue Hills Reservation | AY854064 | AY645054 | AY665773 | AY780939 | AY879117 |
| *Chalciporus piperatus* | MB 04-001 | USA, Massachusetts, Rutland State Park | DQ822797 | DQ534648 | DQ534679 | — | GU187690 |
| *Cheimonophyllum candidissimum* | AFTOL-1765 | USA, Washington, Hazel Wolf Wetlands, King County, near Issaquah | DQ486687 | DQ457654 | DQ435812 | DQ470831 | GU187760 |
| *Chondrostereum purpureum* | AFTOL-441 | USA, Alaska | DQ200929 | AF518607 | AF082851 | AY218477 | — |
| *Climacodon septentrionalis* | AFTOL-767 | USA, Massachusetts, Worcester, Clark University | AY854082 | AY684165 | AY705964 | AY780941 | AY885151 |
| *Coniophora arida* | FP-104367 | USA, Maryland, Near Hwy 329, old farmhouse, south edge of Easton | GU187510 | GU187573 | GU187622 | GU187775 | GU187684 |
| *Coniophora arida v. suffocata* | MUCL30844 | Belgium, Heverlee | GU187511 | GU187568 | GU187623 | GU187779 | GU187685 |
| *Coniophora cerebella* | HK’8’ | Norway | GU187513 | GU187569 | GU187625 | GU187776 | GU187687 |
| *Coniophora marmorata* | MUCL31667 | Belgium | GU187515 | GU187571 | GU187626 | GU187780 | GU187688 |
| *Coniophora olivacea* | FP-104386 | USA, Maryland, Plant Industry Log Cabin woods | GU187516 | GU187572 | GU187627 | — | GU187689 |
| *Coniophora prasinoides* | FP-105969 | USA, Maryland, College Park | GU187519 | GU187576 | GU187621 | GU187785 | GU187691 |
| *Coniophora puteana* | MUCL1000 | Germany | GU187521 | GU187578 | GU187631 | GU187778 | GU187693 |
| *Coniophora* sp. | Braz-6 | Brazil: Sao Paulo, Sao Paulo Station | GU187517 | GU187575 | GU187628 | GU187784 | GU187697 |
| *Coprinus comatus* | AFTOL-626 | USA, California, Alameda Co., Berkeley, UC-Berkeley campus | AY854066 | AY635772 | AY665772 | AY780934 | AY881026 |
| *Cortinarius iodes* | AFTOL-285 | USA, Massachusetts, Rutland State Park | AF389133 | AY702013 | AY771605 | AY536285 | AY881027 |
| *Cotylidia* sp*.* | AFTOL-700 | China, Beijing Province, Dongling Mountains north of Beijing | AY854079 | AY629317 | AY705958 | AY883422 | AY885148 |
| *Echinodontium tinctorium* | AFTOL-455 | Canada | AY854088 | AF393056 | AF026578 | AY218482 | AY885157 |
| *Fibulorhizoctonia* sp. | AFTOL-576 | USA, Louisiana | AY854062 | AY635779 | AY654887 | AY885161 | AY879115 |
| *Flammula alnicola* | AFTOL-1501 | USA, Tennessee, Great Smoky Mountain National Park, Newfound Gap | DQ486703 | DQ457666 | DQ113916 | DQ472714 | GU187699 |
| *Fomitopsis pinicola* | AFTOL-770 | USA, California, Eureka | AY854083 | AY684164 | AY705967 | AY786056 | AY885152 |
| *Gautieria otthii* | AFTOL-466 | Canada | AF377073 | AF336249 | AF393043 | AY218486 | AY883434 |
| *Gomphidius roseus* | AFTOL-1780 | Germany, Bavaria, Siegenburg | DQ534570 | DQ534669 | DQ534682 | GU187818 | GU187702 |
| *Gymnopaxillus nudus* | OSC_18269 | Australia |  | AY177265 |  |  |  |
| *Gymnopaxillus nudus* | OSC_2232 | Australia |  | AY177266 |  |  |  |
| *Gymnopaxillus vestitus* | OSC_1855 | Australia |  | AY177264 |  |  |  |
| *Gyrodon lividus* | REG Gl1 | Germany, Bavaria, Regensburg | DQ534568 | AF098378 | DQ534681 | GU187786 | GU187701 |
| *Gyrodontium sacchari* | MUCL40589 | Africa, Zimbabwe, Matabeleland North, Victoria Falls National Park | GU187522 | GU187579 | GU187632 | GU187764 | GU187703 |
| *Gyroporus* sp. | AFTOL-1518 | Australia, Queensland | EU718107 | EF561627 | DQ534680 | FJ536643 | GU187704 |
| *Hebeloma velutipes* | AFTOL-980 | USA, California, Mendocino County, Caspar Little Lake Road, Caspar | AY818351 | AY745703 | AY752972 | DQ472718 | GU187707 |
| *Hydnellum geogenium* | AFTOL-680 | Norway, Oppland, Lunner | DQ218304 | AY631900 | AY752971 | DQ408133 | DQ059053 |
| *Hydnomerulius pinastri* | MD-312 | USA, Wisconsin, Madison, Madison plot | GU187523 | GU187580 | GU187633 | GU187787 | GU187708 |
| *Hydropus* cf. *scabripes* | AFTOL-535 | USA, Massachusetts, Rutland State Park | DQ404389 | DQ411536 | DQ444855 | DQ457634 | — |
| *Hygrophoropsis aurantiaca* | AFTOL-714 | Germany, Bavaria, Siegenburg | AY854067 | AY684156 | AY662663 | AY786059 | AY883427 |
| *Inocybe dulcamara* | AFTOL-482 | Finland | DQ221106 | AY700196 | AY657016 | AY803751 | DQ435791 |
| *Jaapia argillacea* | CBS252.74 | The Netherlands, Baarn, Groeneveld | GU187524 | GU187581 | AF518581 | GU187788 | GU187711 |
| *Leptosporomyces raunkiaerii* | HHB-7628 | USA, Michigan, Big Bay, Canyon Lake Trail, Huron Mt Club | GU187528 | GU187588 | GU187640 | GU187791 | GU187719 |
| *Leptosporomyces septentrionalis* | JS16122 | Norway | GU187497 | GU187664 |  |  |  |
| *Leucoagaricus barssii* | AFTOL-1899 | USA, California, Berkeley | DQ911600 | DQ911601 | GU187658 | DQ911602 | GU187722 |
| *Leucogyrophana arizonica* | RLG-9902 | USA, Arizona, Mt Bigelow, Santa Catalina Mts, Coronado Natl Forest | GU187527 | GU187582 | GU187636 | GU187792 | — |
| *Leucogyrophana lichenicola* | DAOM194172 | Canada, Ontario | GU187531 | GU187583 | GU187638 | GU187789 | GU187715 |
| *Leucogyrophana mollusca* | L-10277 | USA, Idaho | GU187525 | GU187584 | GU187634 | GU187817 | — |
| *Leucogyrophana montana* | 2998a | Finland | — | GU187665 | — | — | — |
| *Leucogyrophana olivascens* | HHB-11134 | USA, Wisconsin, Madison, University of Wisconsin Arboretum | GU187532 | GU187587 | GU187639 | GU187790 | GU187717 |
| *Leucogyrophana romellii* | T-547 | Canada, Ontario, Nipissing District, Algonquin Prov. Park | GU187529 | GU187586 | GU187635 | GU187794 | GU187720 |
| *Macrolepiota dolichaula* | AFTOL-481 | China, Yunnan, Kunming | DQ221111 | DQ411537 | AY771602 | DQ385886 | DQ435785 |
| *Marasmius rotula* | AFTOL-1505 | USA, New Hampshire, Dixville Notch State Wayside Baby Flume Picnic Area, Coos County | DQ182506 | DQ457686 | DQ113912 | DQ474118 | GU187723 |
| *Megacollybia platyphylla* | AFTOL-560 | USA, Tennessee, Great Smoky Mountain NP | DQ249275 | AY635778 | AY786053 | DQ385887 | DQ435786 |
| *Mycena amabilissima* | AFTOL-1686 | USA, Washington, Hazel Wolf Wetlands, King County | DQ490644 | DQ457691 | DQ457647 | DQ474121 | GU187727 |
| *Mycena aurantiidisca* | AFTOL-1685 | USA, Washington, Indian Creek Trail, Chelan County | DQ490646 | DQ470811 | DQ457694 | DQ474122 | GU187728 |
| *Mycetinis alliaceus* | AFTOL-556 | Russia, Caucasia | AY854076 | AY635776 | AY787214 | AY786060 | AY883431 |
| *Paragyrodon sphaerosporus* | MB 06-066 | USA, Iowa, Iowa City, Hawkeye Court, Mormon Handcart Park | GU187540 | GU187593 | GU187642 | GU187803 | GU187737 |
| *Paxillus vernalis* | AFTOL-715 | China, Beijing Province, Dongling Mountains north of Beijing | DQ267128 | AY645059 | AY662662 | — | DQ457629 |
| *Peniophorella praetermissa* | AFTOL-518 | Germany, Tuebingen | AY854081 | AY700185 | AY707094 | AY787221 | AY885150 |
| *Phaeomarasmius proximans* | AFTOL-979 | USA, Washington, Bogachiel Road, Olympic Peninsula, Jefferson County | DQ404381 | AY380410 | AY752970 | AY333314 | DQ028592 |
| *Phlebopus portentosus* | REG Php1 | South Africa | DQ534569 | AF336260 | DQ534687 | GU187801 | GU187735 |
| *Physalacria bambusae* | AFTOL-515 | Japan | DQ097367 | DQ097349 | AY705953 | DQ474123 | GU187732 |
| *Piloderma fallax* | S-12 | Finland | GU187535 | GU187591 | GU187644 | GU187797 | GU187738 |
| *Pisolithus arrhizus* | REG 588 | New Zealand | GU187538 | AF336262 | DQ534688 | GU187798 | — |
| *Podoserpula pusio* | AFTOL-1522 | Australia, Perth | DQ494688 | DQ470821 | — | GU187804 | — |
| *Porothelium fimbriatum* | AFTOL-1725 | Belgium | DQ490626 | DQ457673 | DQ444854 | DQ472721 | — |
| *Porphyrellus porphyrosporus* | AFTOL-1779 | Germany, Bavaria, Regensburg | DQ534563 | DQ534643 | DQ534689 | GU187800 | GU187734 |
| *Pseudomerulius aureus* | FP-103859 | USA, Maryland, Beltsville, North Woods, BPI | GU187534 | GU187590 | GU187643 | GU187799 | GU187731 |
| *Pseudomerulius curtisii* | REH8912 | Australia, Queensland, Atherton Shire, Danbullah Forest, Danbullah Frorest Drive (Lake Tinaroo), Kauri | GU187533 | GU187589 | GU187641 | GU187796 | GU187725 |
| *Punctularia strigosozonata* | AFTOL-1248 | USA, Wisconsin, Madison | DQ398598 | AF518642 | AF518586 | DQ381843 | DQ408147 |
| *Rhizopogon nigrescens* | MB 06-070 | USA, Massachusetts, Cape Cod, Wellfleet, Marconi Station Rd near Atlantic White Cedar Swamp | — | GU187594 | GU187646 | GU187806 | GU187744 |
| *Rhodocollybia maculata* | AFTOL-540 | USA, Massachusetts | DQ404383 | AY639880 | AY752966 | AY787220 | DQ061279 |
| *Serpula lacrymans* | REG 383 | The Netherlands | GU187542 | GU187596 | GU187649 | GU187809 | GU187752 |
| *Serpula lacrymans* | SL1 | Norway | GU196448 | AM076559 | HM135766 | HM135742 | GU196518 |
| *Serpula lacrymans* | SL5 | Germany, Berlin | GU196451 | AJ440939 | AJ440945 | HM135743 | GU196521 |
| *Serpula lacrymans* | SL198 | Japan, Asahikawa | GU196372 | HM135656 | HM135767 | HM135744 | GU196454 |
| *Serpula lacrymans* | SL199 | Japan, Asahikawa | GU196373 | HM135657 | HM135768 | HM135745 | GU196455 |
| *Serpula lacrymans* | CZ1 | Czech Republic, Jihlava | HM022435 | HM135696 | HM135771 | HM135748 | HM135546 |
| *Serpula lacrymans* | CZ2 | Czech Republic, Jihlava | HM002436 | — | HM135772 | HM135749 | HM135547 |
| *Serpula lacrymans* var. *shastensis* | SHA8-1 | USA, California, Mount Shasta | AM494515 | HM135656 | HM135769 | HM135746 | HM135544 |
| *Serpula lacrymans* var. *shastensis* | SHA30-2 | USA, California, Mount Shasta | HM002472 | HM135657 | HM135770 | HM135747 | HM135545 |
| *Serpula himantioides* | SH16/MUCL30528 | Belgium, Louvain-la-Neuve | GU187545 | GU187600 | GU187651 | GU187808 | GU187748 |
| *Serpula himantioides* | SH136/RLG-12941 | USA, Nevada, G Tunnel, Madison Drift Nuclear test site | GU187547 | GU187602 | GU187654 | GU187811 | GU187750 |
| *Serpula himantioides* | SH17 | Canada, British Colombia, Aleza Lake | AM076494 | AM076527 | HM135773 | HM135750 | HM135548 |
| *Serpula himantioides* | SH20 | Canada, British Columbia, Cowichan Lake | AM076492 | AM076526 | — | HM135751 | AJ557440 |
| *Serpula himantioides* | SH99 | Germany, Bursfelde | AM076514 | AM076547 | AJ440948 | HM135752 | HM135549 |
| *Serpula himantioides* | SH26 | Zimbabwe, Manicaland, Chimanimani area, Muguzo Forest Research Station area | AM076506 | AM076539 | HM135774 | HM135753 | HM135550 |
| *Serpula himantioides* | SH18 | Canada, British Colombia, Mt Revelstoke | AM076499 | AJ557417 | HM135775 | HM135754 | AJ557439 |
| *Serpula himantioides* | SH113 | Norway | AM076497 | AM076530 | HM135776 | HM135755 | HM135551 |
| *Serpula himantioides* | SH164/HHB17587 | USA, Alaska, North side of Kenai Lake, Kenai Peninsula | AM076493 | AF518648 | AF518589 | DQ366283 | DQ059046 |
| *Serpula himantioides* | SH133 | Canada, British Columbia, Snooke | AM076511 | AM076544 | HM135777 | HM135756 | HM135552 |
| *Serpula himantioides* | SH168 | Chile, Concepción | HM135649 | HM135699 | HM135780 | HM135759 | HM135554 |
| *Serpula himantioides* | SH169 | Chile, Concepción | HM135650 | HM135700 | HM135781 | HM135760 | — |
| *Serpula himantioides* | SH129 | China, Yunnan, Chu Xiong, Zi Xi Shan Nature Reserve | HM135651 | HM135701 | HM135782 | — | — |
| *Serpula himantioides* | SH130 | China, Yunnan, Chu Xiong, Zi Xi Shan Nature Reserve | HM135652 | HM135702 | HM135783 | HM135761 | — |
| *Serpula himantioides* | SH115 | China, Yunnan, Chu Xiong, Zi Xi Shan Nature Reserve | HM135653 | HM135703 | HM135784 | HM135762 | HM135555 |
| *Serpula himantioides* | SH127 | China, Yunnan, Chu Xiong, Zi Xi Shan Nature Reserve | HM135654 | HM135704 | HM135785 | — | — |
| *Serpula himantioides* | SH141 | USA, Colorado, Bears Ears Ranger District, Routt Nat'l Forest | HM135655 | HM135705 | HM135786 | HM135763 | HM135556 |
| *Serpula incrassata* | DAOM170590 | Canada | GU187541 | GU187595 | GU187652 | — | GU187751 |
| *Serpula incrassata* | SINC / MUCL 30791 |  | HM135647 | AY491672 | HM135778 | HM135757 | HM135553 |
| *Serpula incrassata* | Si14 /  DAOM 53527 | Canada, British Columbia, Vancouver Island, Victoria | HM135648 | HM135698 | HM135779 | HM135758 | — |
| *Serpula similis* | MUCL43280 | Africa, Cameroon, Dja Biosphere reserve | GU187546 | GU187601 | GU187653 | GU187812 | GU187724 |
| *Serpula pulverulenta* | CBS311.54 | Denmark | GU187543 | GU187597 | GU187650 | — | GU187753 |
| *Serpulomyces borealis* | L-8014 | USA, Washington, Olympic Peninsula, Olympic National Park, Hoh Ranger Station | GU187512 | GU187570 | GU187624 | GU187782 | GU187686 |
| *Simocybe serrulata* | AFTOL-970 | USA, Massachusetts, Mt. Holyoke | DQ494696 | AY745706 | DQ465343 | DQ484053 | GU187755 |
| *Stereum hirsutum* | AFTOL-492 | USA, Massachusetts, Purgatory Chasm | AY854063 | AF393078 | AF026588 | AY218520 | AY885159 |
| *Strobilomyces floccopus* | AFTOL-716 | USA, Massachusetts, Rutland State Park | AY854068 | AY684155 | AY662661 | AY786065 | AY883428 |
| *Stropharia ambigua* | AFTOL-726 | USA, Washington, Hazel Wolf Wetlands, King Co. | AY818350 | AY646102 | DQ092924 | DQ484054 | GU187756 |
| *Suillus bresadolae* | REG 394 | Germany, Bavaria, Regensburg | GU187544 | GU187598 | GU187648 | GU187810 | GU187746 |
| *Suillus pictus* | AFTOL-717 | USA, Massachusetts, Moore State Park | AY854069 | AY684154 | AY662659 | AY786066 | AY883429 |
| *Tapinella atrotomentosa* | REG 78/97 | USA, Massachusetts, Concord | GU187549 | GU187603 | GU187655 | GU187813 | GU187757 |
| *Tapinella panuoides* | MB 05-019 | USA, New Hampshire, White Mountains | GU187548 | GU187604 | GU187657 | — | — |
| *Xerula radicata* | AFTOL-561 | France, Rhone-Alps | DQ241780 | AY645051 | AY654884 | AY786067 | — |
