# Supplementary figures and images for: Evolutionary history of Serpulaceae (Basidiomycota): molecular phylogeny, historical biogeography and evidence for a single transition of nutritional mode
Source: BMC Evol Biol. 2011 Aug 4;11:230. doi: 10.1186/1471-2148-11-230 (PMC3199774; doi:10.1186/1471-2148-11-230)

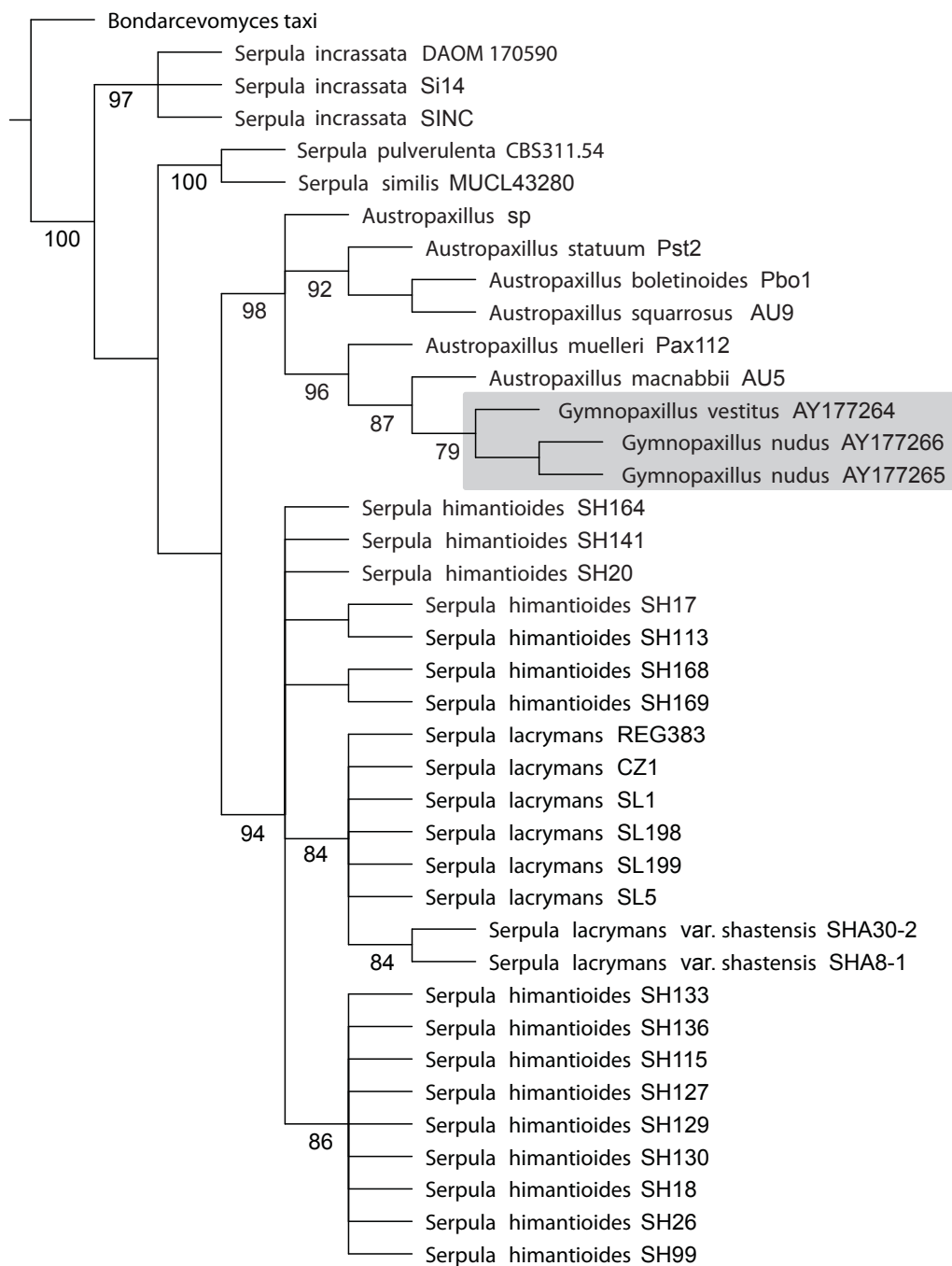

Supplement: Additional file 7 — Serpulaceae nrLSU phylogeny including Gymnopaxillus. Strict consensus tree from a maximum parsimony analysis of the nuclear ribosomal LSU region of 38 Serpulaceae taxa. Bondarcevomyces taxi was used as outgroup. The 16 most parsimonious trees (MPTs) were 311 steps long and had a rescaled consistency index of 0.90 and homoplasy index of 0.06. Jackknife support values (2000 replicates) are superimposed on the branches, showing that the included Gymnopaxillus spp. (grey shading) are nested within Austropaxillus with high support. [file 1471-2148-11-230-S7.PDF]
